# Robust Kalman Filtering Cooperated Elman Neural Network Learning for Vision-Sensing-Based Robotic Manipulation with Global Stability

**DOI:** 10.3390/s131013464

**Published:** 2013-10-08

**Authors:** Xungao Zhong, Xunyu Zhong, Xiafu Peng

**Affiliations:** Department of Automation, Xiamen University, South Siming Road, Xiamen 361005, China; E-Mails: zhongxungao@163.com (X.Z.); pengxiafu@126.com (X.P.)

**Keywords:** visual servoing, dynamic Jacobian estimation, Kalman filtering, Elman neural network, global-state-space

## Abstract

In this paper, a global-state-space visual servoing scheme is proposed for uncalibrated model-independent robotic manipulation. The scheme is based on robust Kalman filtering (KF), in conjunction with Elman neural network (ENN) learning techniques. The global map relationship between the vision space and the robotic workspace is learned using an ENN. This learned mapping is shown to be an approximate estimate of the Jacobian in global space. In the testing phase, the desired Jacobian is arrived at using a robust KF to improve the ENN learning result so as to achieve robotic precise convergence of the desired pose. Meanwhile, the ENN weights are updated (re-trained) using a new input-output data pair vector (obtained from the KF cycle) to ensure robot global stability manipulation. Thus, our method, without requiring either camera or model parameters, avoids the corrupted performances caused by camera calibration and modeling errors. To demonstrate the proposed scheme's performance, various simulation and experimental results have been presented using a six-degree-of-freedom robotic manipulator with eye-in-hand configurations.

## 1. Introduction

Visual sensors integrated with robotic manipulators can be increasingly beneficial for robotic perception and behavioral flexibility in unstructured environments [1]; such sensors have caught much attention, and have applications in all walks of life [2–5].

Vision-based robotic manipulation depends mainly on visual information feedback to control the positioning or motioning of a manipulator [6]. There are two general categories of manipulation: position-based visual servoing (PBVS) and image-based visual servoing (IBVS). There is also a hybrid of the two techniques [7]. These different categories arise from differential definitions of visual feature and feedback control information.

The PBVS vision usage provides information to regulate the end-effector pose (relative to the object in the Cartesian space). Owing to its characteristic of global asymptotic stability, this method is suitable for most industrial robotic manipulators [8]. However, the servoing task is inevitably related to the tedious affine model which is associated with world coordinates, camera frame, and robot base coordinates. Consequently, PBVS is more sensitive, with respect to the camera and object modeling errors [9], and to the possibility of disappearing image features [10–12].

In IBVS, there is direct control of the feature points on the image plane for robotic manipulation, and the image Jacobian matrix is used to describe the differential relation between the image error and the end-effector pose [13]; such direct control has simplified PBVS computation. However, IBVS has its intrinsic drawbacks. Consequently, modified IBVS method have recently mainly focused on: (1) the dynamic visual servo control law, which was proposed to improve poor dynamic performances due to either low vision acquisition frequency or to the time latency processing system [14]; (2) image frame path planning as an effective solution for making camera calibration and robotic kinematics modeling errors more robust [15–17] (such optimization techniques are selected for minimizing both end-effector trajectories in Cartesian space and features trajectories on image planes [18]); and (3) redundant and cooperative 2D visual servo systems [5] and intelligent hybrid schemes [19], which solve typical problems, such as features extraction errors and task singularities, *etc*.

With respect to modified methods, the presented image Jacobian matrix requires depth information and camera calibration parameters (as in traditional IBVS methodologies); such requirements inevitably lead to task singularities, thus making it difficult to ensure the stabilized convergence of a desired target [20]. The Jacobian identification is considered to be a dynamic parameter estimation problem. Solutions to this issue are broadly grouped into two categories.

One category comprises of online estimation techniques (e.g., the famous Broyden-based method and its modified variants) [21,22]. However, these estimation techniques may actually depend on the system configurations and the tasks to be accomplished. Moreover, these techniques are ill-suited for dealing with system environmental noises. In [23], an M-estimator was applied to the Jacobian estimation task, which does not require the model and system parameters. The first literature discussing the Kalman-Bucy filter (KBF) on state space for online Jacobian estimation is proposed in [24]. Those methods have the same properties as recursive least squares-based estimation processing's. However, the Jacobian estimation task may involve singularity instability in global space; failure to have end-effector positioning in cases of large displacement between the initial and desired poses is also a risk.

Another solution to this estimation problem involves machine learning techniques [25,26], which are based on biologically inspired approaches, such as neural networking. These approaches enables the machine to learn the kinematic relationship of manipulators during action-perception cycles [27,28]. While in IBVS leaning techniques, the Jacobian is presented by a trained nonlinear network [25], this approach approximates convergence to the desired position; reaching levels of high servoing precision is difficult because network training is an incomplete convergence process and is sensitive to training samples.

In this paper, the discussion will focus on non-parameter Jacobian matrix estimation problems. A new global-state-space IBVS scheme, which associates KF and ENN learning techniques, is proposed for uncalibrated model-independent robotic manipulation that has robust stability in global-state-space, where the image features are constrained on the camera field-of-view (FOV). The proposed scheme is different from traditional PBVS methods; IBVS methods possess the merits of not needing calibration of either camera parameters or the robotic kinematics model. Moreover, for IBVS methods, Jacobian dynamic estimation does not require depth information. The main contributions of the paper are as follows:

(1) Jacobian online identification problems were solved by introducing state-space infrastructure, which has been incorporated into robust KF techniques. The traditional KF is a minimum-variance state estimator for linear dynamic systems with Gaussian white noises sequence. In most practices, however, the observation noise is compound and the noises are statistically correlated noises (as to being simple white noise sequences). Therefore, we have derived a robust KF estimator with multiple orders of noise for the Jacobian online estimation task.
(2) The KF is sensitive with respect to the initial robotic state and the initial noises' statistical characteristics (*i.e.*, a small perturbation of noise characteristics to dynamic modeling will lead to serious positioning error). For this problem, the ENN was adopted as a global estimator for Jacobian learning, and then the global map relationship between vision space and the robot workspace is represented by the ENN. Notice that this learning method is an approximation of the Jacobian matrix, with respect to the end-effector desired pose in scale motion space; the approximation error present in the offline learning is due to the limited size of the training data.
(3) In the online testing phase, the precise positioning problem is solved by using a robust KF to improve ENN learning so as to achieve robotic convergence to the desired pose. After, the ENN's weights are updated through re-training using a new input-output data pair vector, which is obtained from the KF cycle to ensure robotic global stability manipulation. Finally, we have designed a novel global-state-space IBVS framework associated with robust KF cooperated ENN learning. In our finding, the image Jacobian matrix is estimated without accounting for camera calibration and modeling error. Our servoing system performs robustly despite outside noises and system destabilization.

The paper is organized as follows: a general description of visual servoing (VS) for uncalibrated model-independent robotic manipulation is presented in the next section. The Jacobian estimation with KF is presented in the Section 3; in that section, we have also derived a robust KF with statistically correlated noises. The ENN for global Jacobian learning and a novel global-state-space IBVS scheme are presented in Section 4. The simulation and experimental results are discussed in Section 5. Section 6 presents our conclusions.

## 2. Visual Servoing to Uncalibrated Model-Independent Robotic Manipulation

**Figure 1.**
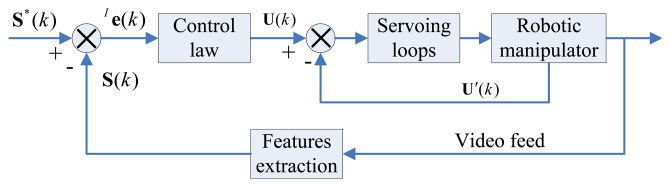
The general structure of uncalibrated model-independent VS system.

In this section, we assume that we have a robotic manipulator, ***R***, that lacks a kinematics model, and where the camera parameters are uncalibrated. As described in Figure 1, uncalibrated model-independent visual servoing (VS) aims to drive the robotic end-effector from the current image feature (**S**(*k*), **S**(*k*) ⊂ **ℜ***^m^*) to the desired image feature (S^*^(*k*), S^*^(*k*) ⊂ **ℜ***^m^*) by using the control variable (**U**(*k*), **U**(*k*) ⊂ **ℜ***^n^*) Generally, a simple local motion of the end-effector will result in the nonlinear complex change of many features on the image plane. The solution for describing the change of features and the robot motion, through the adoption of an image Jacobian matrix, was originally proposed in [13].

The task error **^χ^e**(*k*) in the *n*-dimensional workspace **χ** ⊂ **ℜ***^n^* can be described as:

(1) eχ(k)=PχE(k)−PχT(k):PχE(k)×PχT(k)→ℜn

where **^χ^P***_E_*(*k*) and **^χ^P***_T_*(*k*) represent the end-effector and target pose, respectively.

The *m*-dimensional feature vector on image frame ***I*** is used for task manipulation; image error *^I^***e**(*k*) is given by:

(2) eI(k)=ℏIE(PχE(k))−ℏIT(PχT(k))=S(k)−S*(k):hIE×hIT→ℜm

Where *^I^ħ_E_*, *^I^ħ_T_* belong to the end-effector and target; these variables result from the projection of **^χ^P***_E_*(*k*), **^χ^P***_T_*(*k*) to the image plane, respectively. The Equation (2) is a decision function, when *^I^***e**(*k*) = 0, that meaning is actually equivalent to robot achieving the task manipulation, *i.e.*, **^χ^e**(*k*) = 0.

The association of *^I^***e**(*k*) ⊂ **ℜ***^m^* with the differential changes of the end-effector pose ^χ^**P***_E_*(*k*) ⊂ **ℜ***^n^* is achieved by assuming linearity through the image Jacobian matrix **J**(*k*); this is done through the equation [13]:

(3) e˙I(k)=J(k)⋅P˙χE(k)⇔S˙(k)=J(k)⋅P˙χE(k)

where **J**(*k*) is definedas:

(4) $$\text{J}(k)=\frac{\partial \text{S}(k)}{\partial^{\chi }{\text{P}}_{E}(k)}={\left[\begin{array}{ccc} \frac{\partial s^{1}(k)}{\partial^{\chi }p_{E}^{1}(k)} & \ldots & \frac{\partial s^{1}(k)}{\partial^{\chi }p_{E}^{n}(k)}\\ \ldots & & \ldots \\ \frac{\partial s^{m}(k)}{\partial^{\chi }p_{E}^{1}(k)} & \ldots & \frac{\partial s^{m}(k)}{\partial^{\chi }p_{E}^{n}(k)} \end{array}\right]}_{m\times n}$$

## 3. Jacobian Online Estimation by Introduction in State-Space

### 3.1. The State Equation and Observation Equation

Robot attached visual sensors can be considered an instrumental system whose state vector is formed by concatenations of the row and column elements of the image Jacobian matrix **J**(*k*) [24], *i.e.*,

(5) $$\text{X}(k)={\left[\frac{\partial s^{1}(k)}{\partial^{\chi }{\text{P}}_{E}(k)}\frac{\partial s^{2}(k)}{\partial^{\chi }{\text{P}}_{E}(k)}\ldots \frac{\partial s^{m}(k)}{\partial^{\chi }{\text{P}}_{E}(k)}\right]}^{\text{T}}$$

where $\frac{\partial s^{i}(k)}{\partial^{\chi }{\text{P}}_{E}(k)}={\left[\frac{\partial s^{i}(k)}{\partial^{\chi }p_{E}^{1}}\frac{\partial s^{i}(k)}{\partial^{\chi }p_{E}^{2}}\ldots \frac{\partial s^{i}(k)}{\partial^{\chi }p_{E}^{n}}\right]}^{\text{T}}(i=1,2,3,\ldots ,n)$.

The robotic state-space model is a linear discrete-time dynamical system according to:

(6) X(k+1)=X(k)+W(k)

(7) Z(k)=H(k)X(k)+V(k)

where **X** (*k*) ⊂ **ℜ***^m^*^×^*^n^* is the state vector, **W**(*k*)*^m^*^×n^ is the model noise with zero mean, and variances is **Q** (*k*), **V**(*k*) ⊂ **ℜ***^m^*^×1^ is the observation noise vector, **Z(***k*) ⊂ **ℜ***^m^*^×1^ is the output vector, given by:

(8) $$\text{Z}(k)=\text{S}(k+1)-\text{S}(k)=\text{J}{\left(k\right)}^{\chi }{\dot{\text{P}}}_{E}{\left(k\right)}^{\text{T}}$$

Thus, the observation matrix **H**(*k*) is

(9) H(k)=[p˙χE(k)T0…0p˙χE(k)T]m×mn

The Kalman-Bucy filter (KBF) [29] is a minimum-variance state estimator for linear dynamic systems with Gaussian white noise sequences; the KBF has been used for real-time state estimation [30], as well as for solving the Jacobina matrix online estimation problem [24]. In most practices, however, the observation noises (**V**_(_*_k_*_)_) may be compound, and statistically correlated with model noises (**W**_(_*_k_*_)_), rather than being simple white noise. In the next section, we provide a robust Kalman filtering with statistically correlated noises for image Jacobian estimation task.

### 3.2. Robust KF for Jacobian Estimation

For the universal environment, the observation noise vector (**V**_(_*_k_*_)_) that meets the Markov chain model, given by:

(10) $${\text{V}}_{\left(k\right)}=\psi_{\left(k-1\right)}{\text{V}}_{\left(k-1\right)}+\eta_{\left(k-1\right)}$$

where, **ψ**_(_*_k_*_− 1)_ is the coefficient matrix, **η**_(_*_k_*_)_ is the Gaussian white noise sequence with zero mean, and the variance is **R**_(_*_k_*_)_.

Based upon Equations (6), (7) and (10), we obtain the observation value of system state at *k* + 1 time, as follows:

(11) $$\begin{array}{ll} {\text{Z}}_{\left(k+1\right)} & ={\text{H}}_{\left(k+1\right)}{\text{X}}_{\left(k+1\right)}+{\text{V}}_{\left(k+1\right)}\\ & ={\text{H}}_{\left(k+1\right)}[{\text{X}}_{\left(k\right)}+{\text{W}}_{\left(k\right)}]+\psi_{\left(k\right)}{\text{V}}_{\left(k\right)}+\eta_{\left(k\right)}\\ & =({\text{H}}_{\left(k+1\right)}-\psi_{\left(k\right)}{\text{H}}_{\left(k\right)}){\text{X}}_{\left(k\right)}+\psi_{\left(k\right)}{\text{Z}}_{\left(k\right)}+{\text{H}}_{\left(k+1\right)}{\text{W}}_{\left(k\right)}+\eta_{\left(k\right)} \end{array}$$

Equation (11) can be rewritten as:

(12) $$\underset{{\text{Z}}_{\left(k\right)}^{*}}{\underset{︸}{{\text{Z}}_{\left(k+1\right)}-\psi_{\left(k\right)}{\text{Z}}_{\left(k\right)}}}=\underset{{\text{H}}_{\left(k\right)}^{*}}{\underset{︸}{\left[{\text{H}}_{\left(k+1\right)}-\psi_{\left(k\right)}{\text{H}}_{\left(k\right)}\right]}}{\text{X}}_{\left(k\right)}+\underset{{\text{V}}_{\left(k\right)}^{*}}{\underset{︸}{{\text{H}}_{\left(k+1\right)}{\text{W}}_{\left(k\right)}+\eta_{\left(k\right)}}}$$

Equation (12) can be considered as an equivalent observation equation to replace Equation (7); the statistic characteristics of **W**_(_*_k_*_)_ and V^*^_(_*_k_*_)_ are as follows:

(13) $$\{\begin{array}{l} E[{\text{V}}_{\left(k\right)}^{*}{{\text{V}}_{\left(j\right)}^{*}}^{\text{T}}]=\underset{{\text{R}}_{\left(k\right)}^{*}}{\underset{︸}{\left({\text{H}}_{\left(k+1\right)}{\text{Q}}_{\left(k\right)}{\text{H}}_{\left(k+1\right)}^{\text{T}}+{\text{R}}_{\left(k\right)}\right)}}\delta_{kj}\\ E[\text{W}(k){\text{V}}^{*}{\left(j\right)}^{\text{T}}]=\underset{{\text{S}}_{k}}{\underset{︸}{{\text{Q}}_{\left(k\right)}{\text{H}}_{\left(k+1\right)}^{\text{T}}}}\delta_{kj} \end{array},\delta_{kj}=\{\begin{array}{cc} 1 & k=j\\ 0 & k\neq j \end{array}$$

Equation (13) shows that the observation noise (V^*^_(_*_k_*_)_) is a Gaussian white noise sequence with zero mean, the variance is R^*^_(_*_k_*_)_. V^*^_(_*_k_*_)_ is statistically correlated with the model noise **W**_(_*_k_*_)_, and the cross-covariance matrix is **S**_(_*_k_*_)_. In this case, the application of traditional KF methods was limited. To solve this problem, we introduced a filtering-revise-vector **ρ**_(_*_k_*_)_, such that state Equation (6) can be written as:

(14) $$\begin{array}{ll} {\text{X}}_{\left(k\right)} & ={\text{X}}_{\left(k‐1\right)}+{\text{W}}_{\left(k-1\right)}+\rho_{\left(k-1\right)}\underset{=0}{\underset{︸}{\left({\text{Z}}_{\left(k-1\right)}^{*}-{\text{H}}_{\left(k-1\right)}^{*}{\text{X}}_{\left(k-1\right)}-{\text{V}}_{\left(k-1\right)}^{*}\right)}}\\ & =\underset{\varphi_{\left(k-1\right)}}{\underset{︸}{\left(\text{E}-\rho_{\left(k-1\right)}{\text{H}}_{\left(k-1\right)}^{*}\right)}}{\text{X}}_{\left(k-1\right)}+\rho_{\left(k-1\right)}{\text{Z}}_{\left(k-1\right)}^{*}+\underset{{\text{W}}_{\left(k-1\right)}^{*}}{\underset{︸}{{\text{W}}_{\left(k-1\right)}-\rho_{\left(k-1\right)}{\text{V}}_{\left(k-1\right)}^{*}}} \end{array}$$

In order to eliminate the relevance between W^*^(*k*) and V^*^(*k*), we let $E[{\text{W}}_{\left(k\right)}^{*}{{\text{V}}_{\left(j\right)}^{*}}^{\text{T}}]=0$, *i.e.*:

(15) $$E\left[{\text{W}}_{\left(k\right)}{{\text{V}}_{\left(j\right)}^{*}}^{\text{T}}\right]‐\rho_{\left(k\right)}E\left[{\text{V}}_{\left(k\right)}^{*}{{\text{V}}_{\left(k\right)}^{*}}^{T}\right]=({\text{S}}_{\left(k\right)}-\rho_{\left(k\right)}{\text{R}}_{\left(k\right)}^{*})\cdot \delta_{kj}=0$$

Thus, the filtering-revised-vector is:

(16) $$\rho_{\left(k\right)}={\text{S}}_{\left(k\right)}{{\text{R}}_{\left(k\right)}^{*}}^{-1}$$

Equations (12) and (14) can be considered as a equivalent observation equation and system state equation, respectively. According to the optimal estimation theory [29], the robust Kalman filtering model of statistically correlated noises can be derived as follows:

(1) Estimation equations: the one-step state estimation equation and the variance matrix of estimation error are given by:
  (17) $$\left\{ \begin{array}{ll} {\hat{\text{X}}}_{\left(\text{k}/\text{k}‐1\right)} & ={\hat{\text{X}}}_{\left(k‐1/\text{k}‐1\right)}+\rho_{\left(k‐1\right)}[{\text{Z}}_{\left(k‐1\right)}^{*}‐{\text{H}}_{\left(k\right)}^{*}{\hat{\text{X}}}_{\left(k‐1/\text{k}‐1\right)}]\\ {\text{P}}_{\left(\text{k}/\text{k}‐1\right)} & =E[({\text{X}}_{\left(\text{k}\right)}-{\hat{\text{X}}}_{\left(\text{k}/\text{k}‐1\right)}){\left({\text{X}}_{\left(\text{k}\right)}-{\hat{\text{X}}}_{\left(\text{k}/\text{k}‐1\right)}\right)}^{\text{T}}]\\ & =[\text{E}-\rho_{\left(k‐1\right)}{\text{H}}_{\left(k‐1\right)}^{*}]P_{\left(k-1/k-1\right)}{\left[\text{E}-\rho_{\left(k‐1\right)}{\text{H}}_{\left(k‐\right)}^{*}\right]}^{T}+{\text{Q}}_{\left(k-1\right)}-\rho_{\left(k‐1\right)}{{\text{S}}_{\left(k‐1\right)}}^{\text{T}} \end{array}\right.$$
(2) Updating equations: the state filtering equation, filtering gain, and variance matrix for filtering error are as follows:
  (18) $$\{\begin{array}{ll} {\hat{\text{X}}}_{\left(k/\text{k}\right)} & ={\hat{\text{X}}}_{\left(k/\text{k}‐1\right)}+{\text{K}}_{\left(\text{k}\right)}({\text{Z}}_{\left(k\right)}^{*}‐{\text{H}}_{\left(k\right)}^{*}{\hat{\text{X}}}_{\left(k/\text{k}‐1\right)})\\ {\text{K}}_{\left(k\right)} & ={\text{P}}_{\left(k/k‐1\right)}{{\text{H}}_{\left(k\right)}^{*}}^{\text{T}}{\left[{{\text{H}}_{\left(k\right)}^{*}}^{\text{T}}{\text{P}}_{\left(k/k-1\right)}{{\text{H}}_{\left(k\right)}^{*}}^{\text{T}}+{\text{R}}_{\left(k\right)}^{*}\right]}^{-1}\\ {\text{P}}_{\left(k/k\right)} & =E[({\text{X}}_{\left(\text{k}\right)}‐{\hat{\text{X}}}_{\left(\text{k}/\text{k}\right)}){\left({\text{X}}_{\left(\text{k}\right)}‐{\hat{\text{X}}}_{\left(\text{k}/\text{k}\right)}\right)}^{\text{T}}]\\ & =[\text{I}-{\text{K}}_{\left(k\right)}{\text{H}}_{\left(k\right)}^{*}]{\text{P}}_{\left(k/k-1\right)} \end{array}$$

**Figure 2.**
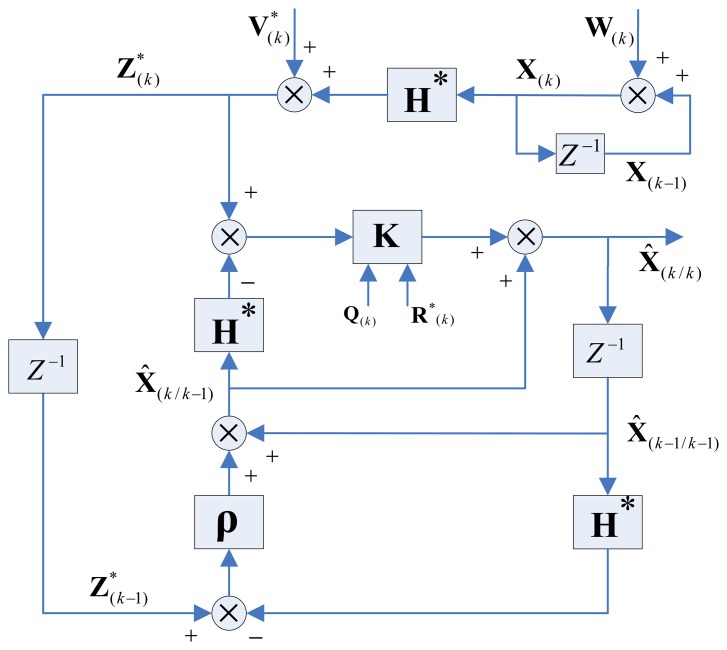
The structure of a robust KF for Jacobian matrix estimation. We introduce a filtering-revise-vector **ρ**_(_*_k_*_)_ to improve the robustness of the filter's performance with respect to universal dynamic noises.

As shown in Figure 2, a robust KF has two basic processes, which are the estimation and the observation updates. The estimation process uses prior-state information and a state model to estimate the current state. The observation update uses the discrepancy between the estimated value and the observed value.

## 4. A Global-State-Space IBVS Scheme for Robotic Manipulation

In this section, a novel global-state-space IBVS method is proposed, which attaches the robust KF Cooperate with Elman neural network learning techniques, so as to enable robot stability manipulation in global-state-space.

### 4.1. Elman Neural Network for Jacobian Approximate Leaning

**Figure 3.**
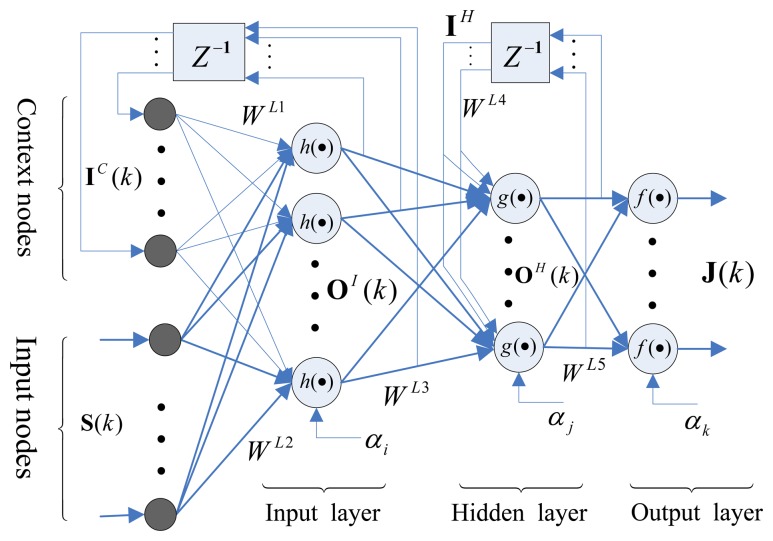
The framework of a feedback Elman neural network with its three layer structure.

The original Elman neural network (ENN) was proposed by Elman in 1990 [31]. ENN is a typical dynamic recurrent network, with particular context nodes (other than the common input layer): the hidden and output layers. ENN's biggest advantage is that its context nodes may be used to memorize the previous activations of the hidden nodes; this makes ENN applicable in the fields of dynamic system identification and prediction control. In this paper, as shown in Figure 3, the framework of feedback ENN with its three layer structure is selected; the framework consists of the input vector **S**(*k*), output vector **J**(*k*), connection weights **W***^Li^* (where *i* = 1, 2, …, 5), the nodal activation functions, *h*(•), *g*(•), *f*(•), and the thresholds, *α_I_*, *α_j_*, *α_k_*, which correspond to the input layer, hidden layer, and output layer, respectively. The mathematical of the ENN is described as follows [32]:

Input layers:
  (19) $$\left\{ \begin{array}{l} {\text{O}}^{I}(k)=h({\text{W}}^{L1}{\text{I}}^{C}(k)+{\text{W}}^{L2}\text{S}(k)-\alpha_{i})\\ {\text{I}}^{C}(k)={\text{O}}^{I}(k-1) \end{array}\right.$$
Hidden layers:
  (20) $$\left\{ \begin{array}{l} {\text{O}}^{H}(k)=g({\text{W}}^{L3}{\text{O}}^{I}(k)+{\text{W}}^{L4}{\text{I}}^{H}(k)-\alpha_{j})\\ {\text{I}}^{H}(k)={\text{O}}^{H}(k-1) \end{array}\right.$$
Output layers:
  (21) $$\text{J}(k)=f({\text{W}}^{L5}{\text{O}}^{H}(k)-\alpha_{k})$$
  where the activation function of input layer *h*(*x*) and hidden layer *g*(*x*) are taken as a sigmoidal function:
  (22) $$h(x)=g(x)=\frac{1}{1+e^{-x}}$$

The output layer activation function *f*(*x*) is taken as a linear function, such that Equation (21) is written as:

(23) $$\text{J}(k)={\text{W}}^{L5}{\text{O}}^{H}(k)-\alpha_{k}$$

The learning algorithm is applied to determine the neural network structure. The goal of training is to obtain the convergence of the connection weights **W***^Li^* (*i* = 1, 2, …, 5) of the input layer to hidden layer, and the hidden layer to output layer. Where 600 samples are used to train the ENN, the input samples to the ENN are the image feature vector (**S**′(*k*) = [**s**_1_(*k*), **s**_2_(*k*), …, **s**_600_(*k*)]_8 × 600_), and the output samples of the network are the Jacobian matrix (**J**′(*k*) = [**x**_1_(*k*), **x**_2_(*k*),…, **x**_600_(*k*)]_48 × 600_). The learning laws with the gradient descent method [32], and the training result (approximately at epoch 107), reach the best validation performances, and the minimum sum squared error (MSE) is 1.3. It is seen that the test output of the network is not very close to the training output, which means that the actual end-effector positioning is approximately close to the desired target; this is due to the approximation error present in offline learning.

### 4.2. Global-State-Space IBVS Scheme

**Figure 4.**
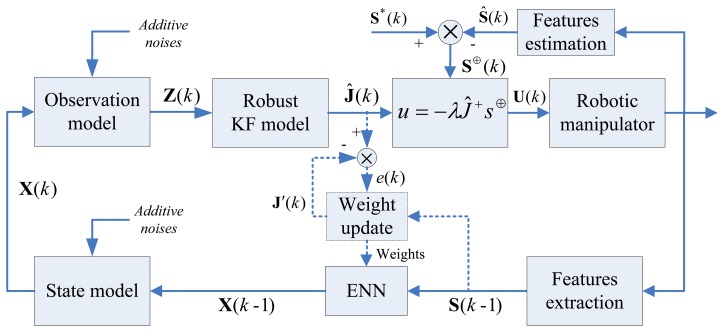
The scheme of global-state-space IBVS, which is attached to a Robust KF Cooperate with ENN learning. Online weights update to ensure robotic global stability manipulation.

The schematic of the proposed global-state-space IBVS is shown in Figure 4. The goal is to extract image features by vision sensor to estimate, online, the desired Jacobian **Ĵ***k*), so as to drive the end-effector to reach the desired pose, as employed by a control law. The scheme consists of the following steps:

(i) First, the Jacobian for the global mapping relationship between the vision space and the robot workspace is approximated using the ENN. As mentioned in last section, it is not necessary to have a very low approximation error during offline training. But in the testing phase, the supposed desired Jacobian **Ĵ**(*k*), with respect to the desired end-effector pose, is estimated by the robust KF (as shown in the dashed box in Figure 4). The ENN's weights were undated and use the gradient descent algorithm to minimize output error *e*(*k*) for a given input image features vector **S**(*k* − 1).
  (24) $$E(k)=\frac{1}{2}e^{\text{T}}(k)e(k)$$
  where *e*(*k*) = (**Ĵ**(*k*) −**J′**(*k*)). The update law of the connecting weight is given by:
  (25) $$w(k+1)=w(k)+\eta e(k)\frac{\partial J^{\prime }(k)}{\partial w}$$
  where *η* is the learning rate.
(ii) The initial state of the robot is very important to robot stability manipulation. The authors in [33] use a fuzzy neural network to provide this initial join guess. In [34], the authors start from the current robot configuration and in [27], a KSOM-based sub-clustering network is used to provide an initial join guess to the inversion algorithm. For the initial Jacobian guess, the common method involves introducing the robotic probe moving at the neighborhood of its initial position *n* times *∂p^j^*^–*n*+1^… *∂p^j^*, and observing the corresponding features displacements *∂p^j^*^–*n*–1^… *∂p^j^* in the image frame. The initial Jacobian matrix could then be obtained reasonably through [24]:
  (26) $$\hat{\text{J}}(0)=[\partial f^{j-n-1}\ldots \ldots \partial f^{j}]{\left[\partial p^{j-n+1}\ldots \ldots \partial p^{j}\right]}^{\text{T}}$$
  In contrast, in this paper, at the initial time *k* = 0, the initial image features **S**(0) captured by the vision sensor are used as an input for ENN. After, the ENN outputs an initial Jacobian guess **J**(0), which can be considered the initial state **X**(0) of the robot. Therefore, our scheme is more flexible since it avoids introducing complex probe motions.
(iii) The robust KF estimator (mentioned in Section 3.2) is used to estimate the desired Jacobian **Ĵ**(*k*) through the following steps:
Step 1robot state initialization as Step (ii).Step 2At *k*−1 time, the approximated Jacobian **J**(*k* − 1) is calculated using Equation (23).Step 3**X**(*k*−1) ←**J**(*k*−1), robot state updating using state Equation (14).Step 4Output vector **Z**(*k*) is obtained using observation Equation (12).Step 5The best state estimate **X**ˆ(*k*) is obtained using the robust KF Equations (17) and (18).Step 6**Ĵ**(*k*) ← **X**ˆ(*k*).Step 7ENN weights update through Equation (25).Step 8*k* −1 ←*k*, go to Step 2.
(iv) The control law should be employed to drive the robot from its present pose to its desired pose. The image error, as shown in Equation (2) at the time instant *k*, is rewritten as:
  (27) $${\text{S}}^{⊕}(k)={\text{S}}^{*}(k)-\hat{\text{S}}(k)$$
  where S*(*k*) is the desired image feature, and **Ŝ**(*k*) = **S**(*k*–1)+ (**S**(*k*–1) –(**S**(*k*–2)) is the estimate value of the image features. Let **^χ^P***_E_*(*k*) = **U**(*k*); then, according to Equation (3), the control law is formed by:
  (28) U˙(k)=P˙χE(k)=‐λJ^+(k)S˙(k)=‐λJ^+(k)S⊗(k)
  where *λ* is the control rate, and **Ĵ**^+^(*k*) is the inverse Jacobian matrix.

## 5. Simulation and Experimental Results

### 5.1. The State Definition of Robotic System

The simulation data consist of four-feature-points, which are used for robot manipulation. The desired features vector S^*^(*k*) does not change over time, and therefore can be calculated before the main control loop of the experiment. On the other hand, the feature vector **S**(*k*) is not constant due to the camera motion and is obtained in each time instant. The features vector is obtained through:

(29) $$\text{S}(k)={\left[\begin{array}{cccc} s_{1} & s_{2} & s_{3} & s_{4} \end{array}\right]}^{\text{T}}={{\left[\begin{array}{llllllll} u_{1} & v_{1} & u_{2} & v_{2} & u_{3} & v_{3} & u_{4} & v_{4} \end{array}\right]}_{8\times 1}}^{\text{T}}$$

where *s_i_* = [*u_i_ ν_i_*]^T^.

Supposing the linear velocity of the end-effector is **V**(*k*) = [*v_x_*, *v_y_*, *v_z_*]**^T^**, the angular velocity is **W**(*k*) = [*w_x_*, *w_y_*, *w_z_*]**^T^**, and the robotic control variable is **U**(*k*) = [**V**(*k*), **W**(*k*)]**^T^**_6 × 1_. According to Equation (4), the 8 × 6 Jacobian matrix is given by:

(30) $$\text{J}(k)=\frac{\partial \text{S}(k)}{\partial \text{U}(k)}={\left[\begin{array}{cccccc} \frac{\partial u_{1}}{\partial v_{x}} & \frac{\partial u_{1}}{\partial v_{y}} & \frac{\partial u_{1}}{\partial v_{z}} & \frac{\partial u_{1}}{\partial w_{x}} & \frac{\partial u_{1}}{\partial w_{y}} & \frac{\partial u_{1}}{\partial w_{z}}\\ \frac{\partial v_{1}}{\partial v_{x}} & \frac{\partial v_{1}}{\partial v_{y}} & \frac{\partial v_{1}}{\partial v_{z}} & \frac{\partial v_{1}}{\partial w_{x}} & \frac{\partial v_{1}}{\partial w_{y}} & \frac{\partial v_{1}}{\partial w_{z}}\\ \dot{\frac{\partial u_{4}}{\partial v_{x}}} & \dot{\frac{\partial u_{4}}{\partial v_{y}}} & \dot{\frac{\partial u_{4}}{\partial v_{z}}} & \dot{\frac{\partial u_{4}}{\partial w_{x}}} & \dot{\frac{\partial u_{4}}{\partial w_{y}}} & \dot{\frac{\partial u_{4}}{\partial w_{z}}}\\ \frac{\partial v_{4}}{\partial v_{x}} & \frac{\partial v_{4}}{\partial v_{y}} & \frac{\partial v_{4}}{\partial v_{z}} & \frac{\partial v_{4}}{\partial w_{x}} & \frac{\partial v_{4}}{\partial w_{y}} & \frac{\partial v_{4}}{\partial w_{z}} \end{array}\right]}_{8\times 6}$$

According to Equation (5), the size 48 × 1 of the system state vector given by:

(31) $$\text{X}(k)={\left[\begin{array}{ccccccccccccc} j_{11} & j_{12} & j_{13} & j_{14} & j_{15} & j_{16} & \ldots & j_{81} & j_{82} & j_{83} & j_{84} & j_{85} & j_{86} \end{array}\right]}_{48\times 1}^{\text{T}}$$

where *j_ik_* refers to the *i*th row and *k*th column of **J**(*k*).

### 5.2. Simulation Evaluation

An eye-in-hand simulation environment was set up to test the performance of the proposed method. Camera movement covers linear, rotational movement, as well as the combination of the translational and rotational movements in the workspace. In simulation, we consider only two difficult tasks, which consist of four cases (combined movement and rotation movement). The evaluation goals of Cases 1 and 2 are test the feature trajectories and the camera trajectory performances of PBVS, IBVS, and our method. Cases 3 and 4 test the performance's global stability and robustness for both KBF and our method.

**Figure 5.**
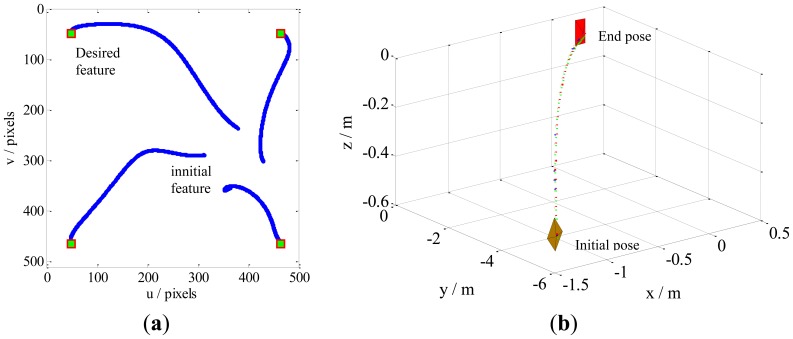
Results obtained by our method for Case 1. The sampling interval is 0.1, and the control rate *λ* is 0.15. Our method does not require camera parameters. The feature trajectories are constrained on the FOV, and the camera trajectory moves in almost a straight line, from the initial pose toward the desired pose. (**a**) The feature trajectories; (**b**) The camera trajectory.

**Figure 6.**
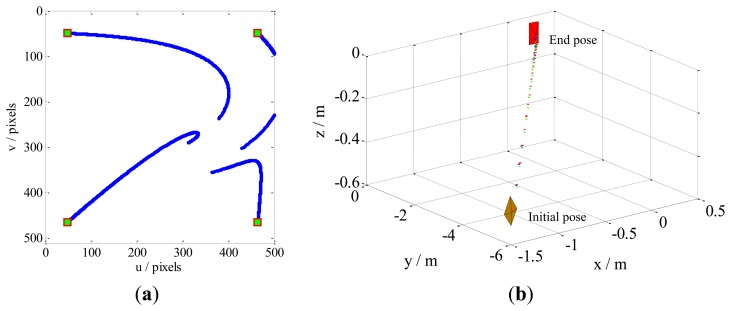
Results obtained by the PBVS method for Case 1. The intrinsic camera parameters are chosen as *u*_0_ = *v*_0_ = 256 and *fk_u_* = *fk_v_* = 1,000. The camera trajectory moves in a seemingly straight line from the initial pose toward desired pose, but the feature trajectories leave the FOV. (**a**) The feature trajectories; (**b**) The camera trajectory.

**Figure 7.**
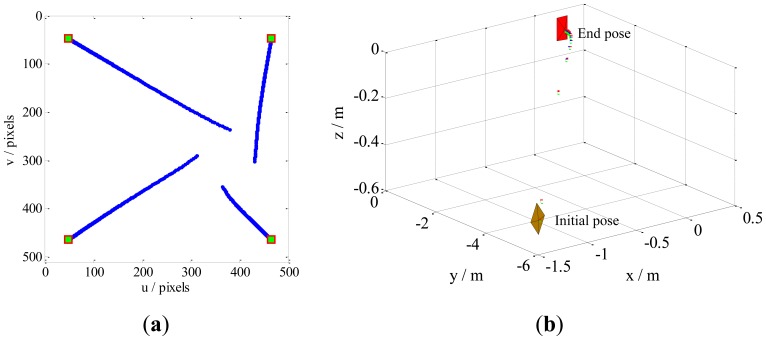
Results obtained by the IBVS method for Case 1. The camera parameters are the same as in the PBVS method. The feature trajectories are constrained on the FOV, but the camera trajectory becomes slightly odd, *i.e.*, the camera moves abruptly. (**a**) The feature trajectories; (**b**) The camera trajectory.

Case 1 involves a combination of the translational and rotational movements of the camera. The results are illustrated in Figure 5a. The feature trajectories on the image plane, obtained through our method, are constrained on the camera field-of-view (FOV), but the PBVS method for this task the feature trajectories easy leaves the FOV (Figure 6a). Additionally, Figure 5b shows that the camera trajectory in the Cartesian space obtained by our method moves on a seemingly straight line from the initial pose toward the desired pose. This result is similar to that obtained by the PBVS method, as illustrated in Figure 6b. The IBVS method for the same task, the feature trajectories are constrained by the controller to move on straight lines from the initial toward desired feature points (Figure 7a), but the camera motion in the Cartesian space (Figure 7b) becomes slight odd, *i.e.*, the camera undergoes an abrupt movement, that is not perfect compared with our method.

**Figure 8.**
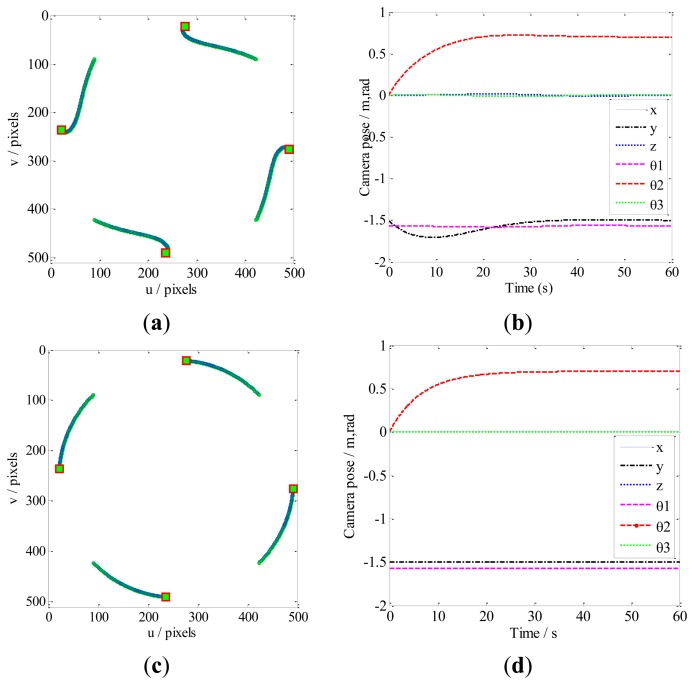
Comparison of our method with the PBVS method for Case 2. The feature trajectories are constrained on the FOV by our method, while the results of PBVS almost leave the FOV. (**a**) Feature trajectories by our method; (**b**) The camera pose by our method; (**c**) Feature trajectories by PBVS; (**d**) The camera pose by PBVS.

Case 2 involves a pure camera rotational movement around the optical axis. The feature points obtained by our method (Figure 8a) move in almost straight lines on the image plane; this movement is very similar to those obtained by the IBVS method. This is because our method is similar to IBVS in that we increase redundant manipulation on the Y-axis to minimize the image feature trajectories (Figure 8b). Additionally, as illustrated in Figure 8c, the PBVS method converge to the desired pose as the image feature points are constrained by the controller to move on circular trajectories. In such circumstances, the feature points almost leave the FOV.

Therefore, compared with the IBVS and PBVS methods, our approach utilizes the advantages of the PBVS method to improve the camera motion trajectory, and takes advantages of the IBVS method to constrain the feature trajectories to avoid situations where features points leave the FOV.

Furthermore, because for PBVS, planar homography is used for pose estimation of the object with respect to the end-effector, and because IBVS is associated with Jacobian computation, both methods are sensitive to camera calibration error. In works [35], the influenceof the calibration parameters on the system is investigated. One solution to this problem uses intelligent hybrid control laws (as proposed in [19]) by introducing neural network reinforcement learning. In our method, the Jacobian is an online estimation, which has nothing to do with camera calibration and system modeling errors. Therefore, our method avoids the corrupted performances caused by calibration and modeling errors.

**Figure 9.**
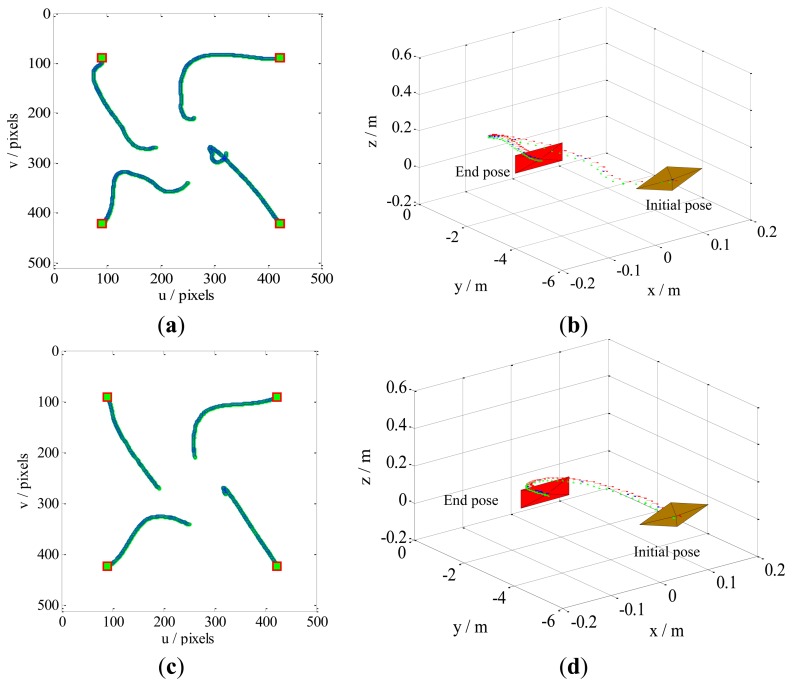
Comparison of our method with KBF for Case 3. The additive noises are set at zero mean, and variances are set at 1 × 10^−3^. (**a**) Features trajectories by KBF; (**b**) Camera trajectory by KBF, camera with slight retreat; (**c**) Feature trajectories by our method; (**d**) Camera trajectory by our method, camera without retreat.

Case 3 deals with dynamic performances in a noisy environment. This case examines the stability of our method, in comparison with the KBF method [21], in a harsh noisy environment. At each sampling instant, uniformly distributed random noises are added to the control system. A Gaussian noise sequence with zero mean, where the variances are 1 × 10^−3^, were added to the state and observation models (the additive noises shown in Figure 4), respectively. The results, shown in Figure 9, are compared with KBF's. In the former, the camera movement and feature trajectories are smoother and more preferable.

**Figure 10.**
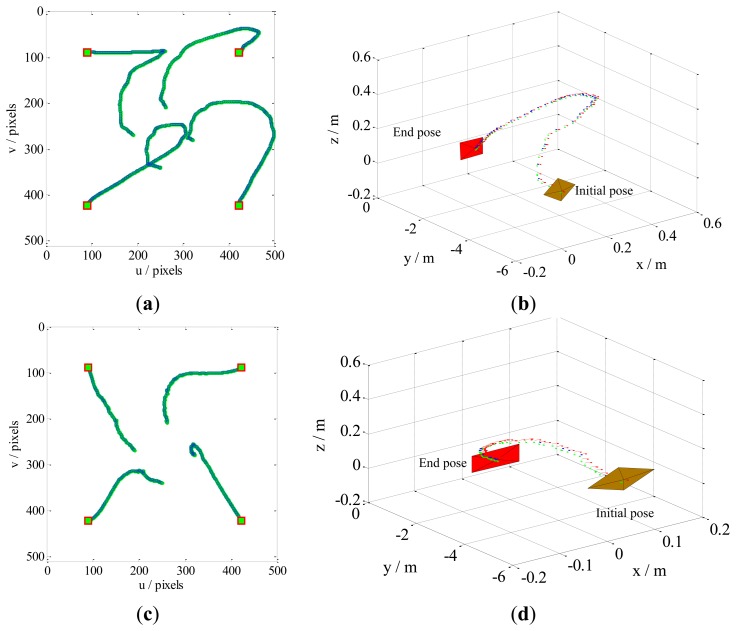
Comparison of our method with KBF for Case 3. The additive noises are set at zero mean, and the variances are set at 9 × 10^−3^. (**a**) Feature trajectories by KBF; (**b**) Camera trajectory by KBF, camera with serious retreat; (**c**) Feature trajectories by our method; (**d**) Camera trajectories by our method, camera without retreat.

When the variance is 9 × 10^−3^, the camera pose in the Cartesian space and the feature trajectories on the image plane for this situation are given in Figure 10. It is obvious that the results of the KBF method are seriously deteriorated by the additive noises; the task goals of reaching the desired position and zeroing the errors are accomplished, but the camera engages in unnecessary retreat, and the image features in the image plane almost leave the FOV. Not only does our method enable convergence on the desired pose, it also robustly performs in the presence of noise. On the other hand, when the noise variances are around 1 × 10^−2^, The KBF method failure to have end-effector positioning, *i.e.*, the camera randomly moves in Cartesian space and the image features in image plan are unknown. In contrast, our method always converges to the desired pose without camera retreat. It is clear that our method has global stability irrespective of the presence of noise.

Case 4 deals with dynamic performances due to system destabilization. During the actual robot manipulation, the statistical characteristics of model noise and observation noise are variational, which leads to system destabilization. For simplicity, but without loss of generality, we consider the statistical parameters **Q**(*k*) and R^*^(*k*) (shown in Figure 2) are classical situation, and then a challenging experiment have been implemented to simulate the robot manipulation, in this paper, a value of **Q**(*k*) = 2^*^(**E**)_48 × 48_ and **R**(*k*) = 0.04^*^(**E**)_8 × 8_ are chosen for the covariance of noises, where **E** is the unit matrix.

**Figure 11.**
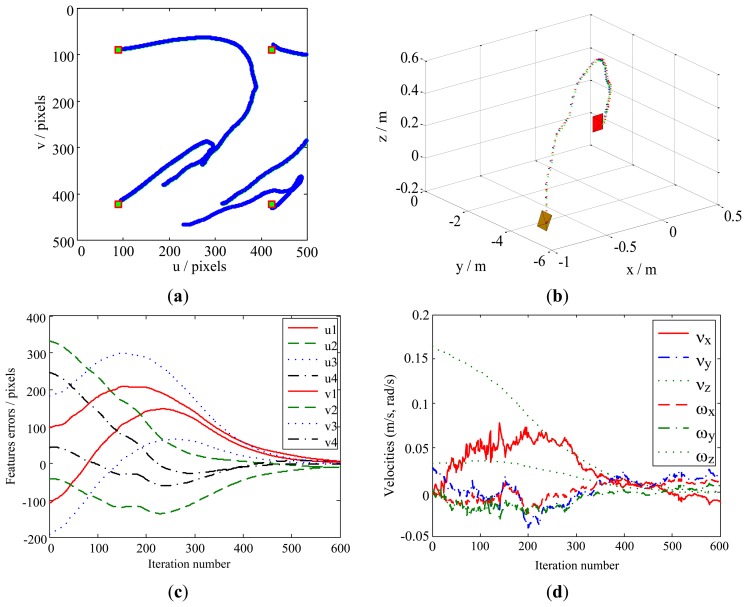
Results obtained by the KBF method for Case 4. (**a**) Feature trajectories; (**b**) Camera trajectory; (**c**) Feature errors; (**d**) Camera velocities.

**Figure 12.**
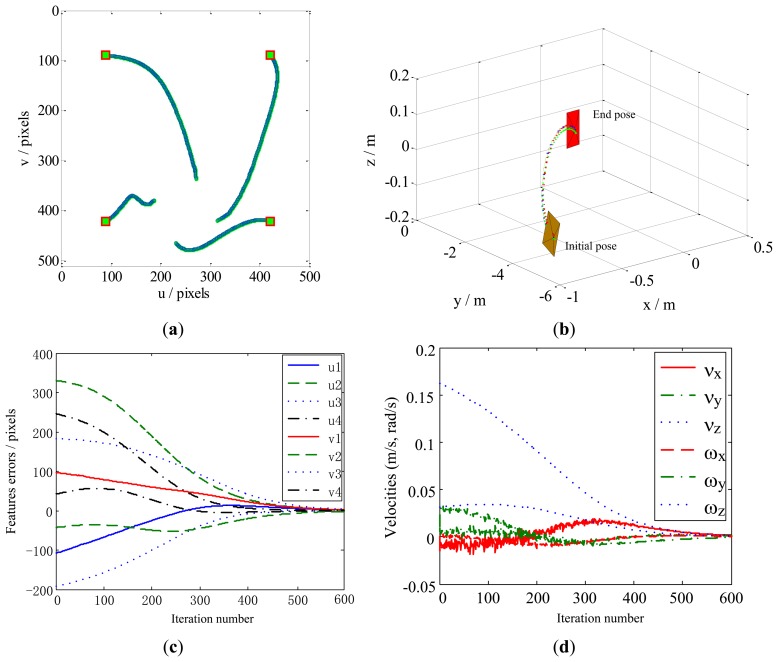
Results obtained by our method for Case 4. (**a**) Feature trajectories; (**b**) Camera trajectory; (**c**) Feature errors; (**d**) Camera velocities.

The dynamic performance of the KBF method is shown in Figure 11. Here, the image features leave the FOV (Figure 11a), and the velocity of the camera does not zero (Figure 11d). As a result, the camera pose fails to converge toward the desired pose at the steady state. For the same case, the performance of our method is presented in Figure 12. It can be seen that the local minima problem of the KBF method has been avoided, and the convergence of both the camera pose and the image features toward their desired targets has been achieved.

On the other hand, different values of Q(*k*) and R(*k*) are considered for different simulations. The results tell us that when the covariance of noises changes slightly, the results of the KBF method will alter largely, and even lose their tracking ability (due to serious camera retreat). When the robust KF was selected to cooperate with ENN learning, our method always worked well, even though the Q(k) and R(k) were changing in a larger region. This means that the proposed global-state-space IBVS is the robust despite system destabilization.

### 5.3. Experimental Results and Discussion

**Figure 13.**
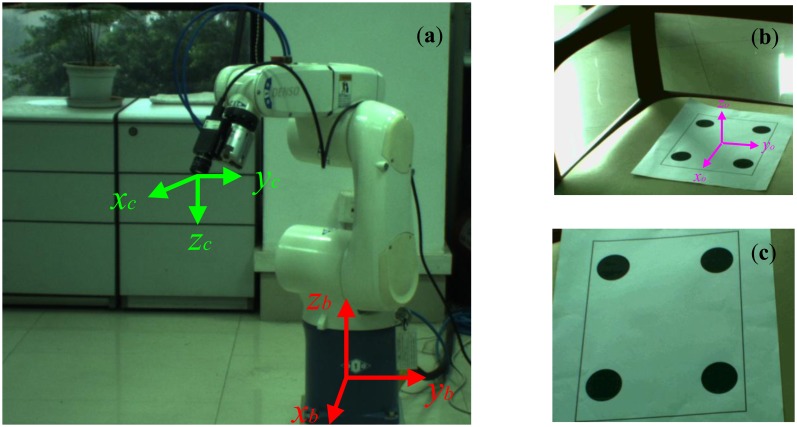
(**a**) Experimental environment with eye-in-hand configurations. (**b**) Initial features. (**c**) Desired features.

The experimental results have been carried out using eye-in-hand configurations (Figure 13a). The task is the online dynamic estimation of a Jacobian J(*k*) so as to drive the end-effector from the initial pose (Figure 13b) to the desired pose (Figure 13c). Our VS system consists of a *DENSO* RC7M-VSG6BA robotic controller, a computer with an Intel Corei5 2.67-GHz CPU, 4GBs of RAM for image processing (The robotic controller and image processing computer can communicate through RS232C serial interface), and a *DENSO* VS-6556GM six-DoF robotic manipulator with a Basler scA1300-32fm/fc camera mounted at its end-effector. The object is an A4 paper with four black-colored small circular disks on it. The object images are captured by the camera at a rate of 30 Hz. The resolution is 640 × 480, and the center points of the small circular disks are used as feature points.

Experiments for the following cases were performed: Case 1 deals with pure rotational movement of the camera, with initial features **S**(*k*) = (481.1 158.9, 244.2 135.2, 220.4 372.1, 457.3 395.8) and desired features S^*^(*k*) = (319.9 19.3, 154.0 190.1, 324.8 356.0, 490.7 185.2). Case 2 deals with translational movement of the camera, with the initial features **S**(*k*) = (217.5 38.1, 89.3 38.1, 89.3 166.3, 217.5 166.3) and desired features S^*^(*k*) = (595.3 202.4, 416.7 202.4, 416.7 381.0, 595.3 381.0). Case 3 deals with a combination of translational and rotational movement of the camera, with the initial features **S**(*k*) = (225.2 299.0, 103.9 286.8, 91.7 408.1, 213.0 420.3) and desired features S^*^(*k*) = (534.8 81.0, 342.9 162.1, 424.0 354.0, 615.9 272.9). The value of *λ* = 0.35 was adopted for the control rate.

**Figure 14.**
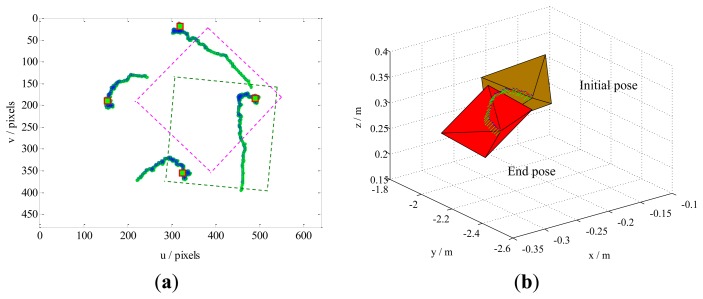

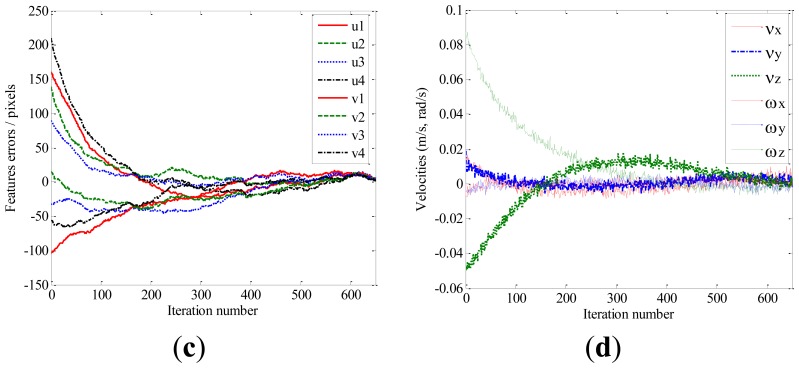
Experimental results obtained by our method for Case 1 rotational movement. (**a**) Feature point trajectories; (**b**) Camera trajectory; (c) Feature errors; (d) Camera velocities.

**Figure 15.**
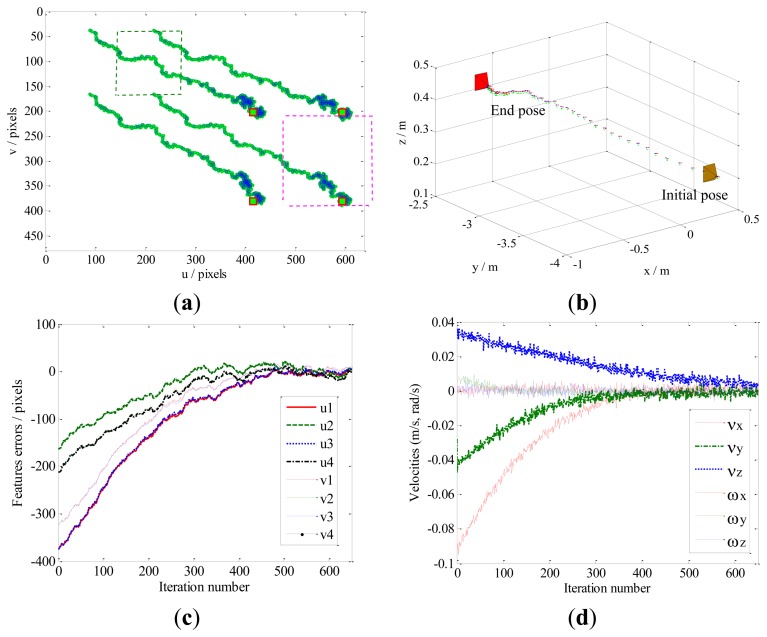
Experimental results obtained by our method for Case 2 translational movement. (**a**) Feature point trajectories; (**b**) Camera trajectory; (**c**) Feature errors; (**d**) Camera velocities.

**Figure 16.**
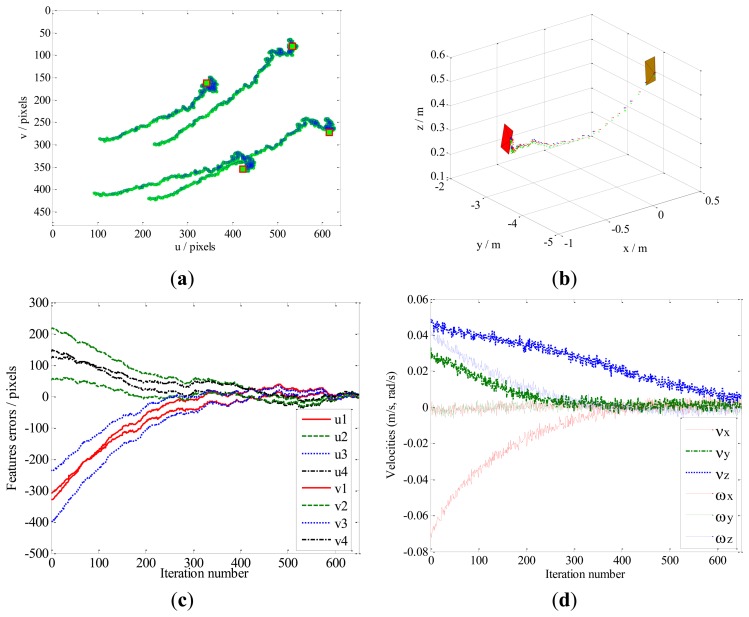
Experimental results obtained by our method for Case 3 translational and rotational movement. (**a**) Feature point trajectories; (**b**) Camera trajectory; (**c**) Feature errors; (**d**) Camera velocities.

The experimental results are shown in Figures 14, 15 and 16. For Case 1 and Case 3, the camera involves rotational movement with low rotational angle values (due to the limitation of the joints angle and the workspace). The results of the image error are minimized, with the initial features converging toward the desired features. The feature trajectories are almost straight lines on the image plane, and the steady-state errors in the image space are in order of 4 pixels for Case 1 and Case 3, while the errors are less than 6 pixels for Case 2. In addition, the camera trajectory in the Cartesian space obtained by our method show that all the tasks of the three cases are completed with global stability without camera retreat. Moreover, the image features remain within the FOV.

In the experiment, our approach direct control of the feature points on the image plane for robotic task manipulation, in other words, the proposed method of observing the changing of features on image plane direct control to the robot converging toward the desired pose with six-degree-of-freedom. As described in Section 2, if the feature errors converge toward zero that meaning is the robot successful achieve the task manipulation. Therefore, the experimental results can validate the proposed method.

## 6. Conclusions

In this work, a new global-state-space IBVS scheme for uncalibrated model-independent robot manipulation in an eye-in-hand configuration is discussed. Here, a robust KF cooperates with ENN learning techniques so as to ensure robust stability in global-state-space with respect to image features within the camera field-of-view (FOV). Also, the image Jacobian matrix is estimated without requiring camera parameters and depth information. Therefore, our method avoids the corrupted performances caused by calibration and modeling errors. Through various simulation and experimental results through IBVS, PBVS, KBF and our method, we have shown that our approach takes the advantages of PBVS to improve the camera moving trajectory, and takes the advantages of IBVS to avoid the loss of image features. Finally, in comparison with the KBF method, our method is robust despite outside and system noises.
